# Targeting Ferroptosis with Small Molecule Atranorin (ATR) as a Novel Therapeutic Strategy and Providing New Insight into the Treatment of Breast Cancer

**DOI:** 10.3390/ph17101380

**Published:** 2024-10-16

**Authors:** Mine Ensoy, Demet Cansaran-Duman

**Affiliations:** Ankara University, Biotechnology Institute, Keçiören, 06135 Ankara, Turkey; mineensoy@gmail.com

**Keywords:** atranorin, small molecule, ferroptosis, breast cancer

## Abstract

**Background/Objectives:** Ferroptosis results from the accumulation of iron-dependent lipid peroxides and reactive oxygen species (ROS). Previous research has determined the effect of atranorin (ATR) on other cell death mechanisms, but its potential for a ferroptotic effect depending on ROS levels is unclear. This study details the therapeutic role of small-molecule ATR through ferroptosis by suppressing MDA-MB-231, MCF-7, BT-474, and SK-BR-3 breast cancer cells. **Methods:** The anti-proliferative effect of ATR on cells was evaluated by xCELLigence analysis, and ferroptotic activity was evaluated by enzymatic assay kits. The changes in gene and protein expression levels of ATR were investigated by the qRT-PCR and western blot. In addition, mitochondrial changes were examined by transmission electron microscopy. **Results:** ATR was found to reduce cell viability in cancer cells in a dose- and time-dependent manner without showing cytotoxic effects on normal breast cells. In BT-474 and MDA-MB-231 cells, ATR, which had a higher anti-proliferative effect, increased iron, lipid peroxidation, and ROS levels in cells and decreased the T-GSH/GSSG ratio. The results revealed for the first time that small-molecule ATR exhibited anti-cancer activity by inducing the glutathione pathway and ferroptosis. **Conclusions:** This study highlights the potential of ATR as a drug candidate molecule that can be used in the development of new therapeutic strategies for the treatment of triple-negative and luminal-B breast cancer subtypes.

## 1. Introduction

Cancer remains a difficult disease to treat and diagnose globally, despite recent advances in treatments. Major challenges for the healthcare system include the rising incidence of cancer and death rates, the lack of long-term treatments, and the side effects of commonly prescribed medicines [1]. Many chemotherapy agents used in routine treatment show efficacy by inducing apoptotic cell death in cancer cells. However, cancer cells develop resistance to apoptosis over time due to their unique metabolism and signaling pathways [2]. It has become an important need to develop effective, alternative treatment strategies to overcome this problem that negatively affects treatment processes.

Breast cancer is the most common cancer worldwide and the leading cause of cancer-related death in women [3,4]. In addition to traditional hormone receptor-positive and -negative types, breast cancer is basically divided into five subtypes with gene expression-based studies using new generation genomic and transcriptomic techniques: luminal A, luminal B, HER2-positive, triple-negative (basal-like, TNBC), and normal-like breast cancer [5]. Breast cancer is a heterogeneous disease with variable biological and morphological characteristics and, therefore, exhibits different clinical behaviors by responding differently to the treatments applied. Despite the advances in diagnosis, prognosis, and treatment processes, an effective treatment method for breast cancer has not been developed, and the incidence of breast cancer is increasing in many countries, especially in developed countries [6]. Therefore, the development of effective methods for the treatment of breast cancer has become a global public health imperative. Traditional treatment methods, including surgery, radiotherapy, hormone therapy, and chemotherapy, which are used in routine treatment, have serious side effects that damage healthy tissues, and patients develop resistance to treatment over time. Considering all these shortcomings, it has become an important need to develop alternative treatment strategies that have fewer side effects and do not induce drug resistance, to elucidate the molecular mechanisms involved in the formation and development of cancer, and to search for effective drug candidates.

It has been known for many years that lichen secondary metabolites can be used as effective alternative small molecules in the treatment of various diseases, especially cancer [7,8]. Atranorin (ATR), one of the important lichen secondary metabolites, is a depside molecule with molecular formula C_19_H_18_O_8_ and chemical name 3-hydroxy-4-methoxy-carbonyl-2,5-dimethylphenyl [9]. ATR has been shown by in vitro, in silico, and in vivo studies to have numerous important biological activities such as anti-proliferative [10], anti-tumor [11], anti-microbial [12], anti-viral [13], anti-inflammatory [14], anti-oxidant [15,16], pro-oxidant [15,16], and anti-cancer [17,18]. In addition, ATR is a redox-active molecule that acts as a pro-oxidant or anti-oxidant agent, depending on the radical [16]. ATR has the potential to be not only an anti-oxidant but also a dose-dependent pro-oxidant in the cell, increasing the production of reactive oxygen species [16]. Although studies have shown that ATR has various biological activities and especially anti-proliferative effects, the molecular characterization of ATR and its effects on cells have not been fully elucidated. The fact that ATR is a potent modulator of the intracellular oxidation system, in addition to its many biological activities, shows that ATR may have anti-proliferative potential by regulating the ferroptosis pathway in cancer cells.

Targeting cell death mechanisms has emerged as a promising therapeutic approach for the treatment of many diseases, including breast cancer. In recent years, research into elucidating the molecular mechanisms involved in the vital processes of cells has increased, and new cell death pathways have been identified. One of the more recently discovered cell death mechanisms is ferroptosis. Ferroptosis is a cell death mechanism that occurs with the accumulation of iron-dependent lipid peroxides and reactive oxygen species and is characterized by morphological changes such as a reduction in cell volume and an increase in the density of the mitochondrial membrane density [19]. Cancer research based on the ferroptosis mechanism has become the focus of attention because targeting the ferroptosis pathway may provide novel therapeutic opportunities in cancer treatment as an alternative to commonly used methods [20,21,22]. To sustain their rapid proliferation and increased bioenergetic and biosynthetic demands, cancer cells require a major reorganization of their metabolism [23]. Ferroptosis becomes a targetable process in cancer cells as a result of this metabolic reorganization, which includes increased lipid peroxides, intracellular iron uptake, and an imbalance in the ferroptosis defense system. In addition, the unique metabolism of cancer cells, the high levels of reactive oxygen species they contain, and the specific mutations they have make some cancers inherently sensitive to ferroptosis. Thus, ferroptosis is an important target for therapeutic intervention in these cancer types [23]. On the other hand, it is known that some cancer cells depend on ferroptosis defense systems to survive under conditions of metabolic and oxidative stress. Disruption of this defense system leads to the death of such cancer cells but does not affect normal cells, allowing targeted therapy [23,24]. Previous studies have shown that ferroptosis has significant potential for use in the treatment of many types of cancer by killing tumor cells and inhibiting tumor growth [25,26]. These findings suggest that ferroptosis, which can be regulated by a small molecule drug candidate, can be used as a new and promising therapeutic strategy for cancer treatment.

The aim of this study was to investigate the effects of ATR on the anti-proliferative effect and ferroptosis pathway of MDA-MB-231 (ER−, PR−, HER2−), BT-474 (ER+, PR+, HER2+), MCF-7 (ER+, PR+, HER2−), and SK-BR-3 (ER−, PR−, HER2+) breast cancer cells. In this study, ATR was found to cause a high level of cell death in MDA-MB-231 and BT-474 breast cancer cells as determined by xCELLigence analysis. Our results demonstrate for the first time that small molecule ATR exerts an anti-proliferative effect by inducing ferroptosis in MDA-MB-231 and BT-474 breast cancer cells, and ATR can be considered as a new therapeutic strategy for breast cancer treatment.

## 2. Results

### 2.1. The Effect of ATR on Breast Cancer Cell Viability

**Figure 1:**
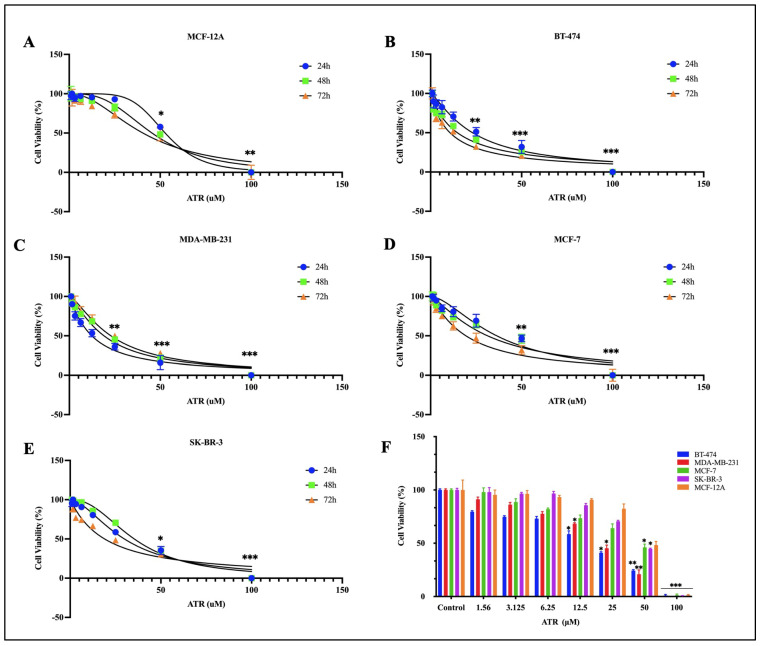
Dose- and time-dependent anti-proliferative effects of ATR on (**A**) MCF-12A, (**B**) BT-474, (**C**) MDA-MB-231, (**D**) MCF-7, and (**E**) SK-BR-3 cells using MTT assays; (**F**) cell viability (%) after 48 h in cells treated with different concentrations of ATR compared to control. Data are represented as the mean ± SD error of three biological replicates. * *p* < 0.05; ** *p* < 0.01; *** *p* < 0.001 (compared to control).

**Figure 2:**
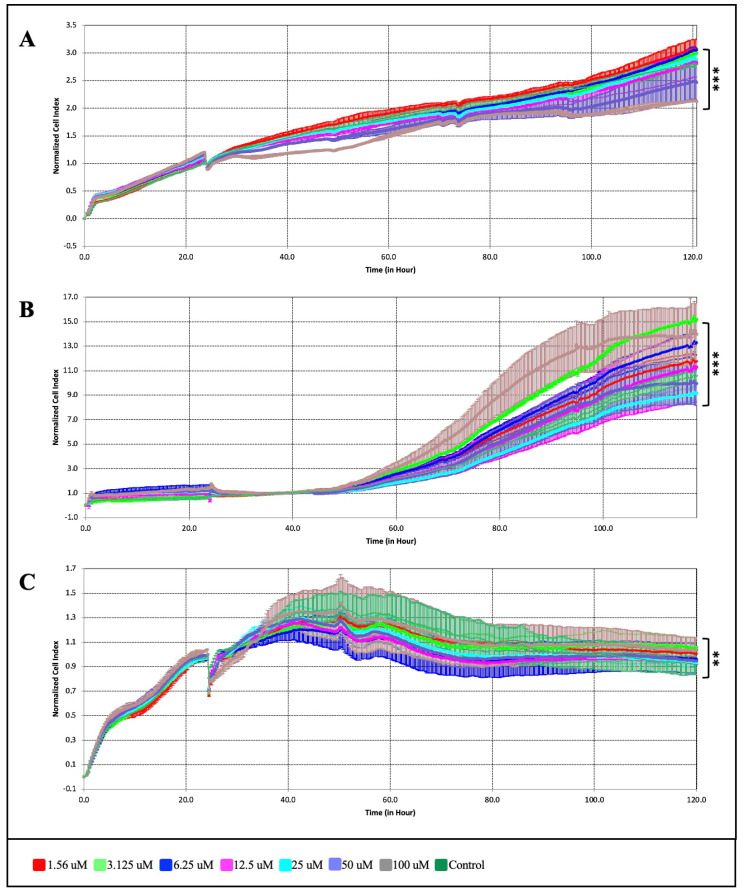
Dose- and time-dependent anti-proliferative effects of ATR on (**A**) BT-474, (**B**) MDA-MB-231, and (**C**) MCF-12A cells using the xCELLigence real-time cell analyzer. (** *p* < 0.01; *** *p* < 0.001).

The anti-proliferative effect of ATR on different subtypes of breast cancer cell lines (MDA-MB-231, MCF-7, SK-BR-3, and BT-474) and on normal breast cells (MCF-12A) was assessed by MTT and xCELLigence RTCA analysis. The results of the MTT and xCELLigence analyses showed that ATR reduced cell viability in breast cancer cells compared to normal breast cells in a dose- and time-dependent manner (Figure 1 and Figure 2). The half maximal inhibitory concentrations (IC_50_) of ATR calculated from the data analysis are listed in Table 1.

According to MTT analysis, ATR suppressed cell viability by % 4.6, 3.7, 6.6, 9.2, 17.6 on MCF-12A normal breast cells at 24, 48, and 72 h at a concentration of 1.56, 3.125, 6.25, 12.5, and 25 μM, respectively, and ATR did not show a cytotoxic effect (*p* < 0.05, *p* < 0.01). However, ATR concentrations higher than 50 μM were found to have a cytotoxic effect on MCF-12A cells at 48 h; the IC_50_ concentration for ATR in MCF-12A cells was 50.21 μM (Figure 1) (*p* < 0.01).

The effect of ATR on breast cancer cells was evaluated, and it was found that ATR showed a higher anti-proliferative effect in BT-474 and MDA-MB-231 cells compared to MCF-7 and SK-BR-3 cells. It was observed that ATR significantly suppressed and inhibited cell growth and viability on BT-474 by 41.44%, 58.97%, 75.62%, and 98.9% (*p* < 0.001). ATR concentrations of 12.5, 25, 50, and 100 µM inhibited MDA-MB-231 cells by 31.66%, 54.71%, 79.06%, and 99.51% (*p* < 0.001). The IC_50_ concentrations of ATR were determined to be 14.70 μM at 48 h on BT-474 cells and 19.03 μM at 48 h on MDA-MB-231 cells (Figure 1B,C). It was found that the IC_50_ concentrations determined for both cell lines had no cytotoxic effects on the normal breast cell MCF-12A at 24, 48, and 72 h. In MCF-7 and SK-BR-3 cells, 1.56, 3.125, 6.25, and 12.5 μM ATR concentrations did not significantly alter cell viability, whereas ATR concentrations above 35–40 μM effectively reduced cell viability in both cell lines (*p* < 0.05). The IC_50_ concentrations of ATR on MCF-7 and SK-BR-3 cells were determined to be 38.71 μM and 38.90 μM at 48 h, respectively (Figure 1D,E). According to MTT analysis, ATR showed no cytotoxic effect on MCF-12A normal breast cells and strongly inhibited MDA-MB-231 and BT-474 breast cancer cells (Figure 1F).

### 2.2. Comparison of the Anti-Proliferative Effect of ATR with Erastin and Ferrostatin-1 in BT-474 and MB-MB-231 Cells by xCELLigence Analysis

The xCELLigence RTCA S16 real-time cell analysis system (Acea Biosciences, San Diego, CA, USA) was used to investigate in detail the anti-proliferative effect of the ATR molecule on MDA-MB-231 and BT-474 breast cancer and normal breast cells. According to the results of the xCELLigence real-time cell analysis, it was observed that ATR had the potential to suppress cell growth in BT-474 and MDA-MB-231 cells as a function of time and dose compared to the untreated sample (Figure 2). BT-474 cell proliferation at 1.56, 3.125, 6.25, 12.5, 25, 50, and 100 µM ATR concentrations was found to be 5.3%, 12.6%, 21.3%, 42.5%, 60.4%, 71.7%, and 93.5%, respectively (Figure 2A) (*p* < 0.01). ATR was found to suppress MDA-MB-231 cell proliferation by 4.11%, 10.2%, 16.4%, 32.9%, 57.9%, 77.2%, and 95.6%, respectively (Figure 2B) (*p* < 0.001). Using RTCA Software Lite (version 2.0), the IC_50_ concentration of ATR on the cells and the effective time interval were calculated, and the IC_50_ concentration was determined to be 14.7 μM in 48 h for BT-474 cells, while it was determined to be 19.0 μM in 48 h for MDA-MB-231 cells (Figure 2B). As a result of the xCELLigence analysis performed with MCF-12A normal breast cells, it was determined that ATR concentrations did not cause a cytotoxic effect compared to the control (Figure 2C) (*p* < 0.01). The IC_50_ concentration of ATR for MCF-12A cells was determined to be 66.7 μM at 48 h (Table 1). The data obtained from the xCELLigence real-time cell analysis and the MTT analysis were found to be compatible.

**Figure 3:**
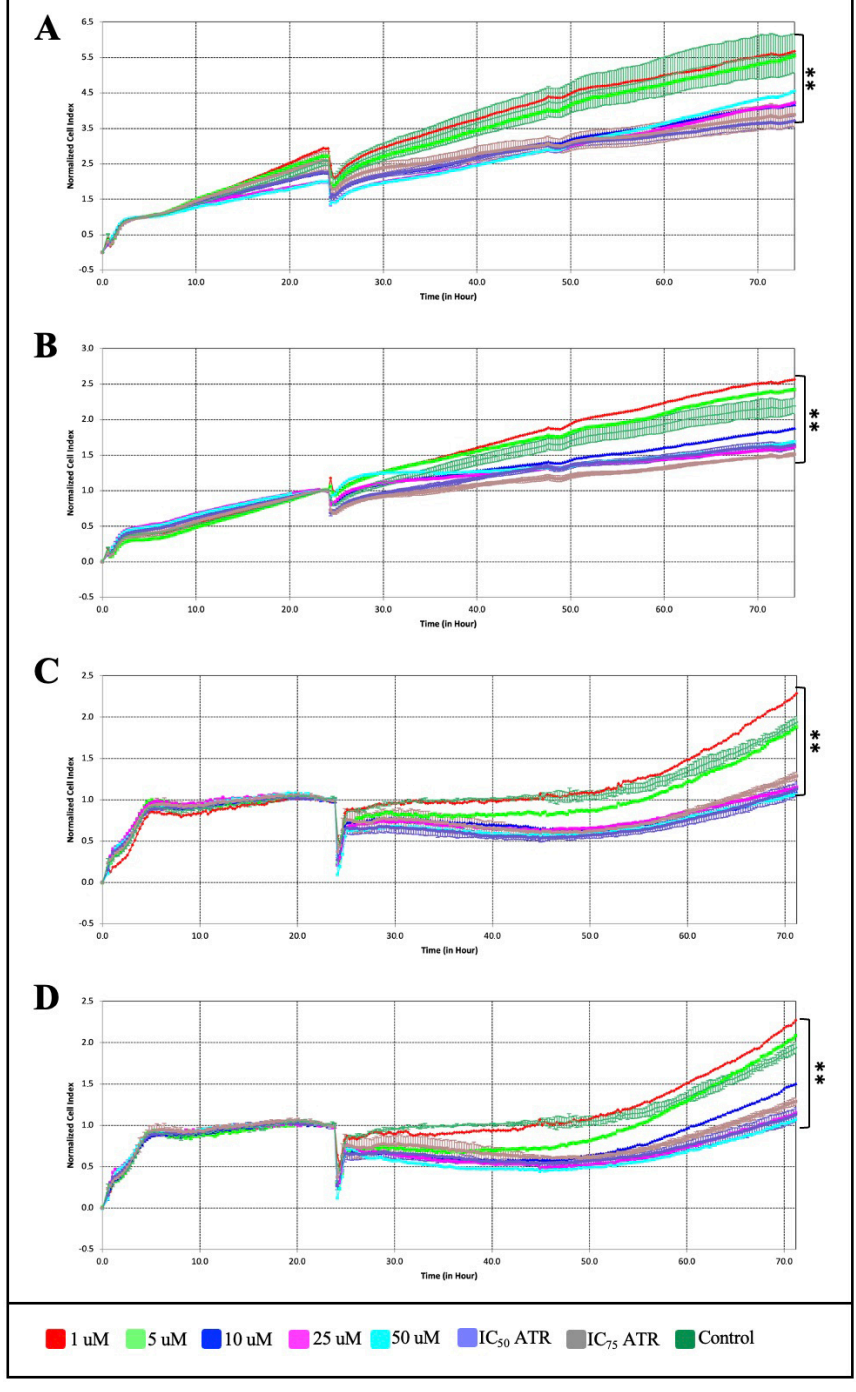
Dose- and time-dependent anti-proliferative effects of (**A**) erastin and (**B**) ferrostatin-1 on BT-474 cells and (**C**) erastin and (**D**) ferrostatin-1 on MDA-MB-231 cells using the xCELLigence real-time cell analyzer (** *p* < 0.01).

**Figure 4:**
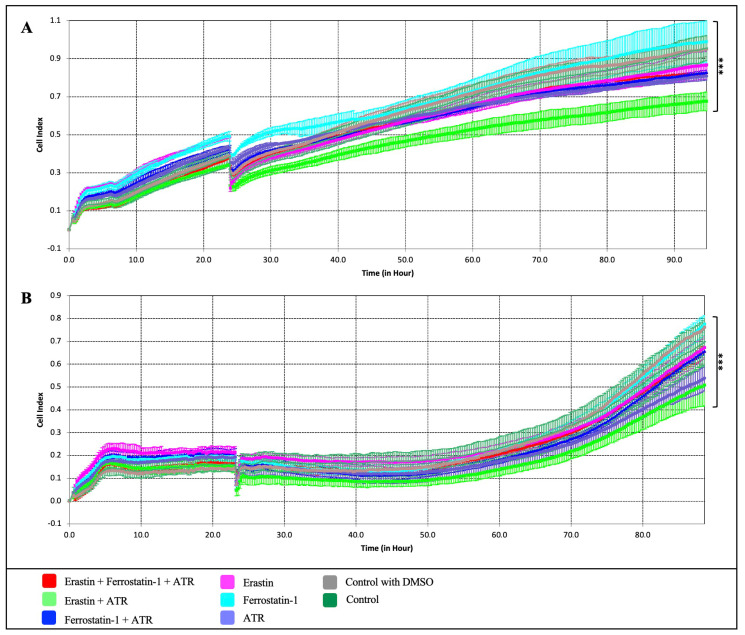
The effect of different combinations of ATR, erastin, and ferrostatin-1 molecules on cell viability and proliferation in (**A**) BT-474 and (**B**) MDA-MB-231 cell lines (*** *p* < 0.001).

To further establish that the growth suppressive effect of ATR on breast cancer cells is mediated through the ferroptosis pathway, ATR was compared with erastin, a small molecule that induces the ferroptosis pathway, and ferrostatin-1, a ferroptosis inhibitor. Erastin and ferrostatin-1 were applied to MDA-MB-231 and BT-474 cells at concentrations of 1, 5, 10, 25, and 50 µM for 72 h using the xCELLigence RTCA system (Figure 3). Firstly, the xCELLigence analysis data showed that the IC_50_ value of erastin was 4.32 μM, ferrostatin-1 was 7.54 μM in BT-474 cells, and the IC_50_ values of erastin and ferrostatin-1 were 3.03 μM and 9.99 μM, respectively, on MDA-MB-231 cells (Figure 3A–D). Secondly, the anti-proliferative effects were investigated by applying different combinations of ATR (IC_50_), erastin (IC_50_), and ferrostatin-1 (IC_50_) to BT-474 and MDA-MB-231 cells for 96 h (Figure 4).

ATR (IC_50_) and erastin (IC_50_) showed anti-proliferative effects on the cells at 53.9% and 59.2% in BT-474 cells and 56.7% and 49.2% in MDA-MB-231 cells, respectively, compared to the control groups; however, ferrostatin-1 was found to increase cell proliferation by 37.8% in BT-474 cells and 28.9% in MDA-MB-231 cells compared to the control (*p* < 0.01) (Figure 4A,B). When ferrostatin-1 was applied together with the ATR molecule, it was observed that cell proliferation decreased by 22.5% on BT-474 cells and 34.2% on MDA-MB-231 cells compared to the control (*p* < 0.01). In both cell lines, it was observed that the suppression of cell proliferation was much higher in the experimental group where ATR (IC_50_) and erastin (IC_50_) molecules were applied together than in the other sample groups (Figure 4A,B). It was found that ATR induces cell death in MDA-MB-231 and BT-474 breast cancer cells, as does the ferroptosis activator erastin, and that this effect is suppressed by the ferroptosis inhibitor ferrostatin-1, indicating that ATR-induced cell death occurs via the ferroptosis pathway.

### 2.3. ATR Increases Intracellular Iron Ion (Fe^+2^) Levels in BT-474 and MDA-MB-231 Cells

**Figure 5:**
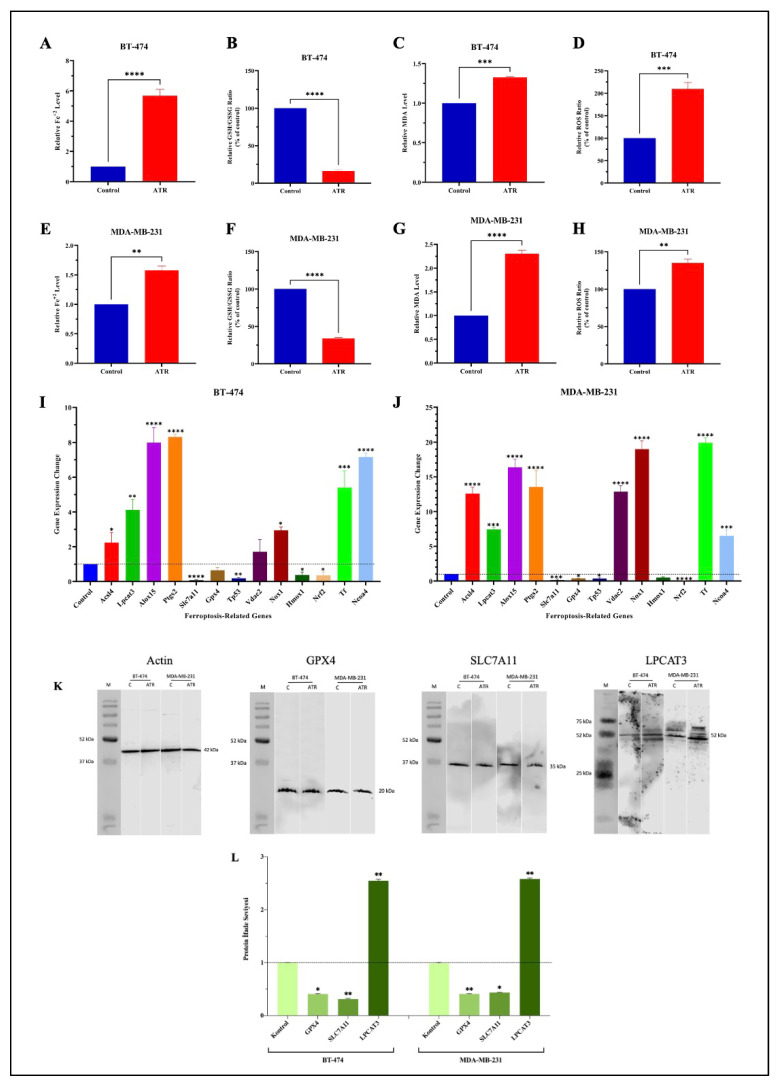
(**A**) Iron ion level, (**B**) T-GSH/GSSG ratio, (**C**) MDA level, (**D**) ROS level in BT-474 cells, and (**E**) iron ion level, (**F**) T-GSH/GSSG ratio, (**G**) MDA level, (**H**) ROS level in MDA-MB-231 cells with and without ATR (IC_50_ concentration); ferroptosis-related gene expression levels in (**I**) BT-474 and (**J**) MDA-MB-231 cells treated and untreated with ATR. *Gapdh* was used as the housekeeping gene. Data are represented as the mean ± SD error of three biological replicates; (**K**,**L**) ferroptosis-related protein levels in BT-474 and MDA-MB-231 cells with and without ATR. Results are normalized to β-Actin. The dash lines are used to show the change according to the control. (* *p* < 0.05; ** *p* < 0.01; *** *p* < 0.001; **** *p* < 0.0001).

The effect of ATR on intracellular iron ion (Fe^+2^) levels in BT-474 and MDA-MB-231 cells was determined using a ferrous iron colorimetric test kit (Elabscience, E-BC-K773-M). Cells were treated with IC_50_ concentration and not treated with ATR for 48 h, and the iron ion level in the cells was determined according to the kit protocol. The results were analyzed as a fold change compared to the control. As a result of the experiment, it was found that there was a 5.7-fold increase in Fe^+2^ levels in BT-474 cells treated with ATR and a 1.6-fold increase in MDA-MB-231 cells compared to the control (Figure 5A,E).

### 2.4. ATR Reduces the Ratio of Total Glutathione (T-GSH)/Oxidized Glutathione (GSSG) in Breast Cancer Cells

According to the results of the analysis, a 4% decrease in T-GSH content was observed in BT-474 cells treated with ATR compared to the control, while an approximately 6-fold increase in GSSG content was determined. Therefore, it was calculated that there was an 83.93% reduction in the T-GSH/GSSG ratio in ATR-treated BT-474 cells compared to the untreated group (Figure 5B). In MDA-MB-231 cells treated with ATR, 5% less T-GSH and 180% more GSSG content were detected compared to the control, and a reduction in the T-GSH/GSSG ratio of 66.18% was calculated (Figure 5F).

### 2.5. ATR Induces Lipid Peroxidation by Increasing the Level of Malondialdehyde (MDA) Levels in Breast Cancer Cells

In BT-474 and MDA-MB-231 cells, an enzymatic test kit (Elabscience, E-BC-K025-M) was used to assess the effect of ATR on the level of intracellular lipid peroxidation by quantifying the amount of malondialdehyde (MDA), the end product of lipid peroxidation. The analysis showed that the level of lipid peroxidation increased 1.3-fold in BT-474 cells treated with ATR compared to untreated cells and increased 2.3-fold in MDA-MB-231 cells (Figure 5C,G).

### 2.6. ATR Induces Reactive Oxygen Species (ROS) in BT-474 and MDA-MB-231 Cells

Intracellular ROS levels were determined in ATR-treated and untreated cells by fluorescence measurement. It was found that the level of ROS in BT-474 cells treated with ATR was increased by 109.7% compared to the control (Figure 5D). In MDA-MB-231 cells, a 35.1% increase in ROS levels was observed in the ATR-treated cells (Figure 5H).

### 2.7. ATR Regulates the Expression Levels of Ferroptosis-Related Genes in BT-474 and MDA-MB-231 Cells

The expression levels of 13 target genes (*Acsl4*, *Lpcat3*, *Alox15*, *Ptgs2*, *Tf*, *Ncoa4*, *Hmox1*, *Slc7a11*, *Gpx4*, *Tp53*, *Nrf2*, *Vdac2*, *Nox1*) were examined to determine the ferroptosis effect of ATR on BT-474 and MDA-MB-231 cells at the transcriptome level by qRT-PCR analysis. As a result of the analysis, the expression level of eight of the ferroptosis-related genes (*Acsl4*, *Lpcat3*, *Alox15*, *Ptgs2*, *Tf*, *Ncoa4*, *Vdac2*, *Nox1*) was increased, while the expression level of five genes (*Hmox1*, *Slc7a11*, *Gpx4*, *Tp53*, *Nrf2*) was decreased in BT-474 cells treated with ATR (IC_50_ concentration) compared to the untreated cells. The relative *Slc7a11* mRNA expression was found to be 13.33 in ATR-treated cells, significantly lower than the control (*p* < 0.0001).

The *Tp53* expression level of ATR-treated BT-474 cells decreased 5-fold (*p* < 0.01), while *Nrf2* and *Hmox1* expression levels decreased approximately 2-fold (*p* < 0.05). The *Ptgs2* gene was found to have the highest expression levels (8.32-fold) in BT-474 cells after ATR treatment (*p* < 0.0001). *Alox15* and *Ncoa4* had an approximately 8-fold increase in gene expression, and *Lpcat3* had an approximately 5-fold increase (Figure 5I) (*p* < 0.0001).

It was found that the expression levels of *Acsl4*, *Lpcat3*, *Alox15*, *Ptgs2*, *Tf*, *Ncoa4*, *Vdac2*, and *Nox1* genes were increased, while the expression levels of *Hmox1*, *Slc7a11*, *Gpx4*, *Tp53*, and *Nrf2* genes were decreased in ATR-treated MDA-MB-231 cells compared to the control. According to the results of the analysis, the genes whose expression level increased the most were *Tf* and *Nox1*, with 19.93- and 19.0-fold changes, respectively (*p* < 0.0001). The expression levels of the genes *Acsl4*, *Ptgs2*, and *Vdac2* also increased by approximately 12-fold, and the expression level of *Alox15* increased by 16.38-fold (*p* < 0.0001). The expression level of the *Nrf2* gene decreased by 11.63-fold compared to the control. It was determined that *Slc7a11* was decreased 6.66-fold and *Gpx4*, *Tp53*, and *Hmox1* were decreased approximately 2-fold compared to the control (Figure 5J) (*p* < 0.05, *p* < 0.001).

### 2.8. ATR Modulates the Expression of Proteins Involved in the Ferroptosis Pathway in BT-474 and MDA-MB-231 Cells

The expression levels of the ferroptosis pathway target proteins Gpx4, Slc7a11, and Lpcat3 were analyzed by western blot assay to determine the ferroptosis effect of ATR on BT-474 and MDA-MB-231 cells at the protein level. β-Actin was used as the housekeeping protein. The target proteins were normalized to β-Actin, and the analysis based on band intensity ratios was performed using the ANOVA method. The results of the western blot analyses showed that BT-474 and MDA-MB-231 cells treated with ATR showed higher levels of Lpcat3 protein expression compared to the control groups and lower levels of Gpx4 and Slc7a11 protein expression compared to the control groups. In BT-474 cells treated with ATR, the expression levels of the proteins Gpx4 and Slc7a11 decreased by 2.3-fold and 3.1-fold, respectively, but the expression level of the protein Lpcat3 protein increased by 2.5-fold compared to untreated cells. In MDA-MB-231 cells, it was determined that the expression levels of Gpx4 increased 2.5-fold, that of Slc7a11 increased 2.3-fold, and that of Lpcat3 decreased 2.5-fold (Figure 5K,L) (Supplementary Figures S1–S7).

### 2.9. ATR Induces Ferroptosis and Causes Cellular Degradation in Breast Cancer Cells

**Figure 6:**
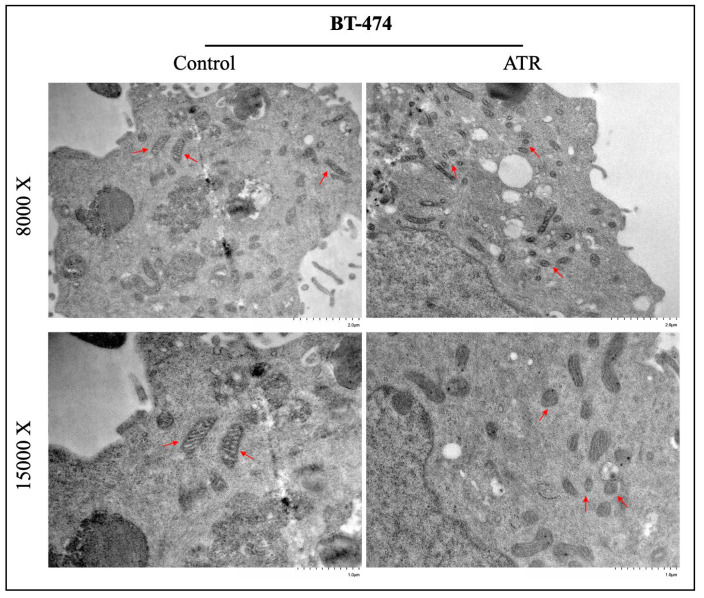
Images of mitochondrial morphology under transmission electron microscopy in BT-474 cells of treated and non-treated ATR. Images were obtained with a magnification scale of 8000× and 15,000×. The red arrows indicate mitochondria.

**Figure 7:**
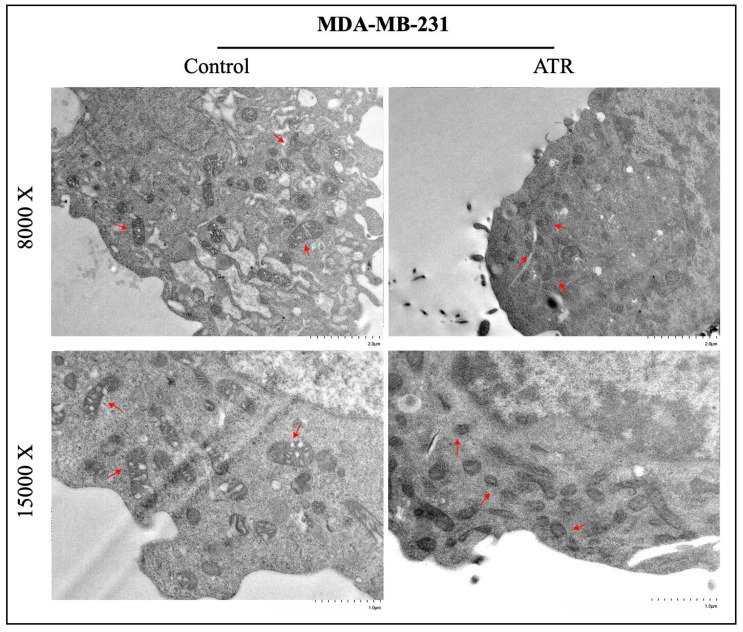
Images of mitochondrial morphology under transmission electron microscopy in MDA-MB-231 cells treated and non-treated ATR. Images were obtained with a magnification scale of 8000× and 15,000×. The red arrows indicate mitochondria.

The change in the ferroptosis effect of treated and untreated ATR on the mitochondrial morphology profile in BT-474 and MDA-MB-231 cells was determined by transmission electron microscopy (TEM). According to the results, ferroptosis-induced changes characterized by the ferroptosis mechanism such as the disappearance of mitochondrial swelling and mitochondrial cristae were detected in BT-474 and MDA-MB-231 cells treated with ATR compared to control groups (Figure 6 and Figure 7).

## 3. Discussion

Breast cancer is a heterogeneous disease with complex molecular mechanisms and different pathological and clinical effects that adversely affect the treatment processes [27]. Despite important studies on the diagnosis and treatment of breast cancer in recent years, an effective treatment method has still not been found. Therefore, the identification of new targets will be important to elucidate the mechanisms underlying the development of the disease, to explore innovative drug candidates, and to develop effective treatment strategies. Ferroptosis is a relatively recently discovered form of regulated cell death characterized by the accumulation of iron-dependent lipid peroxides in the cell, which is morphologically, biochemically, and genetically different from other cell death mechanisms [19,28]. Recent studies have shown that ferroptosis is a promising therapeutic target for the development of new strategies for the treatment of many diseases, especially cancer [29,30,31]. ATR, an important lichen secondary metabolite, has been shown in numerous studies to be an anti-tumor, anti-inflammatory, wound healing, photoprotective, and redox-active molecule [16,18]. However, studies explaining the anti-cancer effect of ATR in breast cancer and its relationship with the ferroptosis mechanism have not been reported so far. In this study, the anti-proliferative effect of ATR, a drug candidate molecule, on breast cancer cells and its relationship with the ferroptosis pathway were investigated. The anti-proliferative effect of ATR through the ferroptosis pathway on breast cancer cells of different subtypes was examined at the cellular, transcriptomic, and proteomic levels. The results showed that ATR inhibited cell viability and proliferation by inducing ferroptosis, thus exhibiting anti-cancer activity in breast cancer cells. The results showed that ATR treatment induced ferroptosis in breast cancer cells compared to the control by increasing iron levels, promoting MDA accumulation, increasing the amount of ROS, ensuring GSH depletion, and also modulating the expression levels of genes and proteins involved in the ferroptosis pathway. While the anti-proliferative effect of ATR was similar to that of erastin, ferrostatin-1 decreased ATR-induced cell death in breast cancer cells. The results of cellular and biochemical analyses, qRT-PCR, and western blot indicated that the suppressive effect of ATR on cell proliferation was related to ferroptosis.

The potential for the use of molecules of natural origin in the treatment of various types of cancer has been investigated for many years, and many natural molecules with significant therapeutic activity have been identified [7,8]. Lichens are organisms that can produce secondary metabolites with a wide range of biological activities, and one of the important secondary metabolites is ATR [7,8]. Studies have shown that ATR has a significant anti-proliferative effect on human ovarian cancer (A2780), human colon cancer (HT-29, LS-174), breast cancer (MCF-7, SK-BR-3, T47D, MDA-MB-231, 4T1), human cervical adenocarcinoma (HeLa), human promyelocytic leukemia, human hepatocellular carcinoma (SK-Hep1, Huh-7, SNU-182), human T cell lymphocytic leukemia, Jurkat, and human melanoma cell (FemX) lines [8,17,18,32,33,34]. Melo et al. have determined through various experiments that ATR is a redox-active molecule that has both pro-oxidant and anti-oxidant properties, as in various natural taxon derivatives [16]. In a study investigating the effect of ATR on liver cancer cell lines (SKHep1, Huh-7, and SNU-182), it was shown that ATR suppressed cell growth, induced cell cycle arrest, and increased cell death. It has also been determined that ATR was also found to cause necrosis-like cell death caused by cell membrane disruption as well as apoptotic death [35]. The effect of ATR as a pro- or anti-oxidant molecule varies depending on the concentration and the type of free radicals in the cells [15,16,35]. However, the mechanism of action of ATR on cells has not yet been fully elucidated. Although there are studies investigating the anti-proliferative effect of ATR on breast cancer cells [17,18,36], there is no comprehensive study investigating the mechanism underlying the therapeutic activity of ATR. This study investigated the effect of ATR, which as a redox-active molecule has pro-oxidant capacity and is known to increase ROS levels in a dose-dependent manner, on ferroptosis in breast cancer cells and showed that ATR has the potential to induce ferroptosis in cells by increasing intracellular ROS and lipid ROS levels and decreasing anti-oxidant system-related gene expression levels.

In recent years, researchers have focused on investigating the induction of ferroptosis using natural drug candidates to develop effective, innovative, and alternative treatment strategies for the treatment of breast cancer [23,25,37]. Erastin is a small molecule called “eradicator of RAS and ST-expressing cells” [38] and has been proven to activate the ferroptosis mechanism by various studies [19,39]. Ferrostatin-1 is a potent inhibitor of ferroptosis that exerts an anti-ferroptotic effect by eliminating lipid peroxides in the presence of reduced iron [40,41]. In studies investigating the effect of drugs or drug candidate molecules on the ferroptosis mechanism, erastin is frequently used as a positive control and ferrostatin-1 as a distinctive inhibitor from other cell death mechanisms [25,42,43,44]. In our study, the anti-proliferative effect of ATR associated with the ferroptosis pathway was compared with erastin and its effect on regulating ferroptosis with ferrostatin-1. Our results showed that the cell proliferation-reducing effect of ATR was significantly similar to erastin and that the ATR-induced anti-proliferative effect was suppressed by ferrostatin-1. The fact that the effect of ATR on cancer cell viability and growth suppression was similar to that of a ferroptosis inducer and that this effect was suppressed by a ferroptosis inhibitor suggests that ATR exerts its anti-proliferative effect through the ferroptosis pathway.

Wei et al. found that eupaformosanin (Eup), a natural compound, significantly inhibited cell viability in TNBC cells. The study showed that Eup increased iron and lipid ROS in cells, induced GSH depletion, and ferrostatin-1 reversed Eup-induced cell death, suggesting that the Eup-induced anti-proliferative effect in TNBC cells may be related to ferroptosis [45]. Similarly, our results showed that ATR increased the iron and lipid ROS levels, decreased the GSH/GSSG ratio, and ferrostatin-1 reduced the anti-proliferative effect of ATR in BT-474 and MDA-MB-231 cells. Another study found that ketamine-induced ferroptosis by reducing the expression of Gpx4, which is involved in glutathione metabolism by catalyzing the reduction of peroxides in breast cancer cells [46]. A recent study showed that tiliroside, a natural flavonoid glycoside, induces the ferroptosis mechanism in TNBC cells by affecting glutathione metabolism [47]. In our study, the GSH ratio decreased significantly after ATR treatment in BT-474 and MDA-MB-231 cell lines, and the expression level of *Slc7a11* and *Gpx4* genes involved in glutathione metabolism decreased significantly. These results suggest that ATR is effective in modulating the glutathione mechanism and suppressing the anti-oxidant system in breast cancer cells, thus contributing to the induction of ferroptosis in cells.

It is known that elevated intracellular iron levels catalyze Fenton reactions, increase ROS generation, and induce ferroptosis [48,49]. Ma et al. showed that the combination of the lysosomotropic agent siramesine, which is known to be effective in inducing cell death in breast cancer, and the kinase inhibitor lapatinib, which is used in the treatment of breast cancer, induces ferroptosis by causing iron accumulation in cells [50]. In our study, *Tf* and *Vdac2* gene expression levels were significantly increased, and *Hmox-1* and *Nrf2* expressions were decreased in ATR-treated BT-474 and MDA-MB-231 cells compared to control. The *Tf* (transferrin) gene is involved in iron transport into the cell by forming a complex with *TfR* (tranferin receptor). The expression of *Tf* is regulated depending on iron levels. *Hmox1* is a cytoprotective enzyme induced in response to cellular stress and has a dual role in ferroptosis, acting as both a pro- and anti-ferroptotic enzyme [51,52]. *Nrf2* is involved in the suppression of ferroptosis by preventing lipid peroxidation and iron accumulation in cells by regulating iron and anti-oxidant system elements [52,53]. Induction of *Tf* and suppression of *Nrf2* are important for induction of the ferroptosis pathway, whereas suppression or induction of *Hmox1* varies according to the redox status of the cell. In addition, according to the results of the biochemical analysis in which the iron load level was determined, it was found that intracellular Fe^+2^ levels were significantly increased in ATR-treated cells compared to the control. On the other hand, our TEM analysis results showed that ATR caused mitochondrial dysfunction in both breast cancer cell lines. It is known that mitochondrial function disorders can impair iron utilization or export and thus lead to cytoplasmic iron accumulation [54]. Our results suggest that ATR induces ferroptosis in BT-474 and MDA-MB-231 cells by regulating the expression levels of genes involved in iron metabolism, causing an increase in the level of intracellular iron load and enhancing ROS generation through catalysis of mitochondrial dysfunction and Fenton reactions.

It is known that lipid metabolism plays an important role in the ferroptosis mechanism and that lipid peroxidation products accumulated in the cell induce ferroptotic cell death [55,56]. In the experimental results, it was determined that the amount of malondialdehyde, which is the end product of lipid peroxidation, increased in BT-474 and MDA-MB-231 cells with ATR application. It was also found that the expression levels of *Acsl4, Lpcat3,* and *Alox15* genes, which are involved in lipid metabolism and play an important role in ferroptosis [55,57], increased in both cell lines, significantly in MDA-MB-231.

BT-474 is in the luminal B subtype of breast cancer, characterized by the expression of the estrogen receptor (ER) and human epidermal growth factor receptor 2 (HER2) [58,59]. MDA-MB-231 lacks ER, progesterone receptor (PR), and HER2 and is in the triple-negative breast cancer (TNBC) subtype [58,59]. In comparison to BT-474, MDA-MB-231 cells have a higher metastatic potential and are more resistant to conventional therapies. The immunopathologic difference between the cells greatly affects the response to treatment [58]. In addition, the cells have different genetic mutations, copy number variations, and different epigenetic modifications. The differences in ferroptosis-related gene expression levels in response to ATR between BT-474 and MDA-MB-231 cells are likely due to the different molecular subtypes, genetic and epigenetic characteristics, cellular redox status, ferroptosis sensitivity, and metabolic profiles of these cell lines.

One of the most important morphologic features that distinguish ferroptosis from other cell death pathways is mitochondrial changes in cells, such as shrinkage of mitochondrial volume, reduction or disappearance of cristae, and increased membrane density [54]. The results of TEM analysis showed that ATR caused mitochondrial changes such as mitochondrial volume shrinkage and reduction in mitochondrial crystals in BT-474 and MDA-MB-231 cells compared to control. It was determined that ATR was effective in inducing mitochondria-mediated ferroptosis in breast cancer cells and had anti-cancer activity by regulating energy metabolism and mitochondrial oxidative stress in cancer cells.

The treatment of breast cancer is mostly based on chemically induced drugs. Conventional chemotherapeutic agents such as doxorubicin or cisplatin often lead to apoptotic cell death by causing DNA damage and increasing free radical production [60]. In contrast, ferroptosis is a non-apoptotic form of cell death driven by iron accumulation and lipid peroxidation. Targeting ferroptosis with drug candidate molecules will be useful in treating cancers that are resistant to apoptosis-based therapies [61]. Conventional chemotherapy drugs often have systemic side effects such as myelosuppression, nephrotoxicity, and cardiotoxicity. Candidate molecules of natural origin that act through different mechanisms have the potential to reduce common side effects and offer a more effective therapeutic index [62,63,64]. ATR, as a naturally derived small molecule therapeutic agent, has a unique mechanism of action and low side effect potential and is, therefore, a promising alternative agent or adjunct to existing breast cancer therapies.

The data obtained showed that ATR significantly suppressed cell viability and cell proliferation in breast cancer cells. Analyses at biochemical, mRNA, and protein levels showed that the glutathione mechanism was suppressed, and lipid peroxidation, ROS, and iron levels were increased as a result of ATR application in BT-474 and MDA-MB-231 cells. Moreover, ATR has been proven to cause mitochondrial dysfunction in the cells. It was also shown that ATR-induced cell death was similar to erastin-induced cell death and was reversed by ferrostatin-1. These results indicate that ATR is an anti-proliferative agent for breast cancer cells and exerts its effect through the ferroptosis mechanism. Further investigation of the effects of ATR in vivo and/or in combination with other ferroptosis inducers will provide a clearer path for clinical application.

## 4. Materials and Methods

### 4.1. Atranorin Molecule

Atranorin (ATR, C_19_H_18_O_8_) was purchased from Cayman Chemical (CAS No: 479-20-9, Ann Arbor, MI, USA). ATR was dissolved in DMSO, and serial dilutions (1.56, 3.125, 6.25, 12.5, 25, 50, and 100 μM) were prepared from the stock solution. Erastin and ferrostatin-1 were commercially available (Cayman, 17754 and Cayman, 17729, respectively, Ann Arbor, MI, USA) and were prepared by dissolution in DMEM medium containing 0.05% DMSO.

### 4.2. Cell Line and Cell Culture

MDA-MB-231, BT-474, MCF-7, and SK-BR-3 breast cancer cells and MCF-12A normal breast cells were obtained from the American Type Culture Collection (ATCC). MDA-MB-231, MCF-7, and BT-474 cells were cultured in Dulbecco’s modified Eagle’s medium (DMEM) (Gibco, 11965092, Merck Life Science, Darmstadt, Germany), and SK-BR-3 cells were cultured in McCoy’s 5A (modified) medium (Gibco, 16600082, Merck Life Science, Darmstadt, Germany) supplemented with 10% fetal bovine serum (FBS) (Gibco, 16000044, Merck Life Science, Darmstadt, Germany) and 1% penicillin-streptomycin (Biological Industries, 03-031-1B, Kibbutz Beit-Haemek, Israel). MCF-12A cells were cultured in DMEM F-12 (HAM) (Biological Industries, 01-0951A, Kibbutz Beit-Haemek, Israel) supplemented with 10% FBS, 10 μg/mL insulin (Humulin, HI 0219, Lilly, Indianapolis, IN, USA), 20 ng/mL epidermal growth factor (EGF) (Gibco, PHG0311, Merck Life Science, Darmstadt, Germany), 0.5 mg/mL hydrocortisone (Sigma-Aldrich, H3160, Darmstadt, Germany), and 1% penicillin-streptomycin at 37 °C in a humidified atmosphere containing 5% CO_2_.

### 4.3. Cell Viability and Cell Death Assays

#### 4.3.1. MTT Method

The anti-proliferative effect of ATR on cells was assessed by the MTT (3-(4,5-dimethylthiazol-2-yl)-2,5-diphenyltetrazolium bromide) assay. The 1 × 10^3^ MDA-MB-231 cells, 5 × 10^3^ MCF-7, SK-BR-3, BT-474, and MCF-12A cells were cultured in 96-well plates, and after 24 h, the cells were treated with different concentrations of ATR (1.56, 3.125, 6.25, 12.5, 25, 50, and 100 μM). The plates were incubated for 24, 48, and 72 h. After incubation, MTT (Serva, 298-93-1, Heidelberg, Germany) at 5 mg/mL was dissolved in PBS, and 20 µL was added to each well and incubated for 4 h. The MTT was then removed from the wells, and 100 µL isopropanol (Sigma-Aldrich, K53323734-119, Darmstadt, Germany) was added to each well. After shaking the plate in the dark for 15 min, the absorbance was measured at 570 nm using a microplate reader (Tecan, Infinite 200 PRO, Männedorf, Switzerland).

#### 4.3.2. xCELLigence Real-Time Cell Analyzer (RTCA) System

The anti-proliferative effect of ATR on MDA-MB-231 and BT-474 breast cancer cells and MCF-12A normal breast cells was analyzed using the xCELLigence RTCA S16 system (Acea Biosciences, San Diego, CA, USA). After background measurement, MDA-MB-231 cells were seeded at 1 × 10^3^ cells/well, and other cells tested were seeded at 5 × 10^3^ cells/well in a 16-well E-plate with 100 μL medium. The plate was placed in the instrument and incubated for 24 h. Different concentrations of ATR (1.56, 3.125, 6.25, 12.5, 25, 50, and 100 μM) were then added to the wells of the E-plate in a volume of 100 µL. The instrument was set to run for 120 h, with readings taken every 15 min, and the proliferation curve was monitored in real-time on the program. IC_50_ concentrations of ATR were obtained using the xCELLigence RTCA software program (version 2.0).

The anti-proliferative effects of Erastin and Ferrostatin-1 on MDA-MB-231 and BT-474 cells were also evaluated using the xCELLigence RTCA S16 system. In the experiment, Erastin and Ferrostatin-1 were applied to the cells at concentrations of 1, 5, 10, 25, and 50 µM, and cell proliferation was assessed with measurements taken every 15 min for 72 h. In addition, BT-474 and MDA-MB-231 cells were treated with different combinations of molecules (Erastin IC_50_ + Ferrostatin-1 IC_50_ + ATR IC_50_; Erastin IC_50_ + ATR IC_50_; Ferrostatin-1 IC_50_ + ATR IC_50_; Erastin IC_50_; Ferrostatin-1 IC_50_; ATR IC_50_; controls (DMSO and DMEM). Cell proliferation was monitored in real-time for 90 h. IC_50_ values were calculated using RTCA Software Lite.

### 4.4. Detection of Reactive Oxygen Species (ROS)

Intracellular ROS levels were assessed using a Reactive Oxygen Species Fluorometric Assay Kit (Elabscience, E-BC-K138-F, Houston, TX, USA) after treatment with ATR and untreated. MDA-MB-231 and BT-474 cells were seeded at 5 × 10^4^ cells/well in 6-well plates. After 24 h incubation, the cells were treated with ATR. After 48 h incubation, the treated and untreated cells were collected and incubated with 20 μM DCFH-DA probe at 37C °C for 60 min according to the manufacturer’s protocol. Microplate readers (Tecan, Infinite 200 PRO, Männedorf, Switzerland) were used for detection at 525 nm for emission and 500 nm for excitation. The percentage of ROS generation in ATR-treated cells was calculated relative to untreated cells.

### 4.5. Measurement of Ferrous Iron (Fe^+2^) Content

Ferrous (Fe^+2^) iron in MDA-MB-231 and BT-474 cells was detected using the Ferrous (Fe^+2^) Iron Colorimetric Test Kit (Elabscience, E-BC-K773-M, Houston, TX, USA) according to the manufacturer’s instructions. Briefly, cells were seeded in petri dishes and allowed to adhere overnight. ATR was applied to the cells at IC_50_ concentrations, and after 48 h, 4 × 10^6^ cells per sample were harvested. The cells were homogenized and incubated with 150 µL of Iron Assay Kit reagent for 10 min at 37 °C. The absorbance was then measured at 593 nm using a microplate reader (Tecan, Infinite 200 PRO, Männedorf, Switzerland). The absorbance value obtained from the test samples (nmol/106) was normalized and calculated with the iron standards obtained from the kit. The ferrous iron (Fe^+2^) level in ATR-treated cells was calculated as the fold change compared to untreated cells.

### 4.6. Measurement of Total Glutathione (T-GSH)/Oxidized Glutathione (GSSG) Ratio

T-GSH and GSSG levels were assessed quantitatively using the T-GSH/GSSG assay kit (Elabscience, E-BC-K097-M, Houston, TX, USA). MDA-MB-231 and BT-474 cells (1 × 10^6^ cells/mL) were seeded into petri dishes. After 24 h, the cells were treated with ATR. Cells were harvested by trypsinization and homogenization using the protein precipitator kit. Half of the homogenized samples were pre-treated to measure the level of GSSG in the cells; the samples were incubated with GSH Scavenger Auxiliary Solution and GSH Scavenger Kit for 60 min at room temperature. A reactive working solution was then added to all samples and incubated for 5 min. Samples were added to a 96-well plate at 50 µL per well, substrate was added to the wells and incubated for 25 min. The products of T-GSH and GSSG were detected using a microplate reader (Tecan, Infinite 200 PRO, Männedorf, Switzerland) with absorption at 412 nm. The relative ratio of T-GSH/GSSG in the cells was calculated by comparing the control with the graph of the standard curve.

### 4.7. Measurement of Lipid Peroxidation

Lipid peroxidation in MDA-MB-231 and BT-474 cells was measured using the malondialdehyde (MDA) test kit (Elabscience, E-BC-K025-M, Houston, TX, USA) using the TBA method. MDA, the result of the lipid peroxidation reaction, directly correlates with the level of lipid peroxidation in the cell. Briefly, cells were seeded in petri dishes at 1x10^6^ cells/mL and incubated for 24 h. The cells were treated with ATR for 48 h. After incubation, the cells were collected and then lysed by sonication. The kit reagents were added to the cell lysates and incubated at 100 °C for 60 min. The samples were cooled to room temperature and added to a 96-well plate. The relative MDA levels in the cells were determined by measuring the absorbance at a wavelength of 532 nm in a microplate reader (Tecan, Infinite 200 PRO, Männedorf, Switzerland). Lipid peroxidation levels in ATR-treated cells were calculated by comparison with untreated cells and the standard curve of MDA.

### 4.8. Total RNA Extraction and cDNA Synthesis

Total RNA was extracted from ATR-treated and untreated MDA-MB-231 and BT-474 cells using TRIzol reagent (Invitrogen™, 15596026, Waltham, MA, USA). The quality and quantity of extracted RNAs were assessed by optical density measurement using a spectrophotometer (NanoDrop, ND-1000, Wilmington, DE, USA). Overall, 1 μg total RNA and the EURx cDNA synthesis kit (NG dART RT Kit, E0801), including oligo(dT) primers, were used for all cDNA synthesis reactions according to the manufacturer’s protocol.

### 4.9. Quantitative Real-Time PCR (qRT-PCR)

The expression level of the ferroptosis-related genes was determined by performing quantitative real-time PCR analysis using 5x HOT FIREPol EvaGreen qPCR Mix Plus (Solis Biodyne, 08-25-00001, Tartu, Estonia) and the Roche LightCycler 96 system (Roche Diagnostics, Rotkreuz, Switzerland). Thermal cycler conditions were as follows: 95 °C for 10 min, 45 cycles of 95 °C for 10 s, 52–58 °C for 45 s, and 72 °C for 10 s. The threshold cycle (Ct) values of the genes were normalized to the housekeeping gene GAPDH, and the fold change in gene expression was quantified by the 2^−ΔΔCt^ method. The sequences of thirteen primers used for qRT-PCR are listed in Supplementary Table S1.

### 4.10. Protein Isolation and Western Blot Analysis

MDA-MB-231 and BT-474 cells treated and untreated with IC_50_ concentrations of ATR were harvested with cold PBS using a cell scraper. Cells were pelleted by centrifugation at 300× *g* for 5 min at 4 °C. The pellet was mixed with lysis buffer containing protease and phosphatase inhibitor cocktails and incubated for 30 min. The samples were centrifuged at 14,000× *g* for 10 min at 4 °C. Sample protein concentrations were determined using BCA protein standards and Bradford reagent. Protein samples were separated by 12% sulphate-polyacrylamide gel electrophoresis (SDS-PAGE) and transferred to polyvinylidene fluoride (PVDF) membranes (Bio-Rad, 1620177, Hercules, CA, USA). The membranes were blocked with 5% non-fat milk for 1 h at room temperature and then exposed overnight at 4 °C to GPX4 (Cell Signaling, 52455, Darmstadt, Germany), LPCAT3 (Proteintech, 678882-1, Rosemont, USA), xCT/SLC7A11 (Cell Signaling, 12691, Darmstadt, Germany), and β-Actin (FineTest, FNab00872, Boulder, CO, USA) primary antibodies at appropriate sample dilutions. After incubation, the membrane was incubated for 2 h at 25 °C with horseradish peroxidase (HRP)-conjugated secondary antibodies, anti-mouse IgG (Cell Signaling, 7076, Darmstadt, Germany) and anti-rabbit IgG (Cell Signaling, 7074, Darmstadt, Germany). Protein bands were visualized with the SuperSignal West Femto Maximum Sensitivity Substrate Kit (Thermo Scientific, 34095) using the Odyssey Infrared Imaging System (LICOR Biosciences, Lincoln, NE, USA). Protein band densities were qualified using ImageJ software (Pierce, Rockford, IL, USA, version 1.8.0), and data were normalized to β-Actin.

### 4.11. Transmission Electron Microscopy (TEM) Study

To investigate the possible morphological changes in mitochondria in BT-474 and MDA-MB-231 cells, we used transmission electron microscopy (TEM). Briefly, BT-474 and MDA-MB-231 cells were seeded in a 6-well plate and treated with IC_50_ concentrations of ATR. Cells were fixed with 2.5% glutaraldehyde in 0.1 M PBS (pH 7.4) overnight at 4 °C. The cells were then treated with 1% osmium tetroxide, a gradient of ethanol dehydration, epoxy resin embedding, and lead citrate staining. The samples were then imaged using a transmission electron microscope (HITACHI, HT7800, Ibarak, Japan).

### 4.12. Statistical Analys

The MTT assay results were analyzed by one-way ANOVA. The xCELLigence real-time cell analysis assay results were analyzed using RTCA Software Lite software (version 2.0). The results of lipid peroxidation level, iron ion level, T-GSH/GSSG ratio, and reactive oxygen species (ROS) level determination experiments performed to determine the ferroptotic effect by enzymatic assays were analyzed by the student *t*-test method. Gene expression levels obtained by qRT-PCR studies were evaluated by the 2^−ΔΔCt^ method and analyzed using the one-way ANOVA method. Band intensities obtained from Western blot results were calculated by the ImageJ program and analyzed by the one-way ANOVA method. All experiments were performed in triplicate, and data were presented as mean ± standard deviation (SD) and analyzed using GraphPad Prism 9.5.1 (GraphPad Software, Inc., San Diego, CA, USA (version 9.5.1.)). For all experiments, the *p*-values shown as * *p* < 0.05, ** *p* < 0.01, *** *p* < 0.001, and **** *p* < 0.0001 were considered statistically significant.

## 5. Conclusions

The cytotoxic effect of ATR, a lichen secondary metabolite, on breast cancer cell lines BT-474 (ER+, PR+, HER2+), MDA-MB-231 (ER−, PR−, HER2−), MCF-7 (ER+, PR+, HER2−), and SK-BR-3 (ER−, PR−, HER2+) of different histological subtypes and on the normal breast cell MCF-12A was determined for the first time using the MTT assay and the xCELLigence RTCA system. The ATR molecule was found to inhibit cell viability and proliferation in breast cancer cells without having a cytotoxic effect on healthy breast cells. The anti-proliferative effect of the ATR molecule was similar to that of erastin, a ferroptosis activator, and this effect was suppressed by ferrostatin-1, a ferroptosis inhibitor. In conclusion, our study revealed for the first time the potential of the ATR molecule as a drug candidate molecule and its use for breast cancer treatment through the ferroptosis mechanism, unlike chemotherapeutics used in routine treatment of breast cancer patients.

## Figures and Tables

**Table 1:** The half maximal inhibitory concentrations (IC_50_) of atranorin (ATR) on cells.

Cell Lines	MTT Assay	xCELLigence Analyzer	Time
BT-474	14.70 μM	14.7 μM	48 h
MDA-MB-231	19.03 μM	19.0 μM	
MCF-7	38.71 μM	-	
SK-BR-3	38.90 μM	-	
MCF-12A	50.21 μM	66.7 μM	

## Data Availability

The datasets used and/or analyzed during the current study are available from the corresponding author on reasonable request.

## Supplementary Materials

The following supporting information can be downloaded at: https://www.mdpi.com/article/10.3390/ph17101380/s1, Table S1: The sequences of ferroptosis-related primers. Figure S1: The raw western blot image of Actin. Figure S2: The raw western blot image of GPX4 in BT-474. Figure S3: The raw western blot image of GPX4 in MDA-MB-231. Figure S4: The raw western blot image of SLC7A11 in BT-474. Figure S5: The raw western blot image of SLC7A11 in MDA-MB-231. Figure S6: The raw western blot image of LPCAT3 in BT-474. Figure S7: The raw western blot image of LPCAT3 in MDA-MB-231.
