# Supplementary material for: Targeting Ferroptosis with Small Molecule Atranorin (ATR) as a Novel Therapeutic Strategy and Providing New Insight into the Treatment of Breast Cancer
Source: Pharmaceuticals (Basel). 2024 Oct 16;17(10):1380. doi: 10.3390/ph17101380 (PMC11509994; doi:10.3390/ph17101380)

**Supplement Table S1.** The sequences of ferroptosis related-primers

| **Primers** | **Forward Sequence 5'-3'** | **Reverse Sequence 5'-3'** |
|---|---|---|
| *Acsl4* | GCTATCTCCTCAGACACACCGA | AGGTGCTCCAACTCTGCCAGTA |
| *Lpcat3* | AGGAAAGATACCAAACAGCATCA | GATAGTCTTCTGTGATGTGGGG |
| *Alox15* | CAGACGTGGCTGTGAAAGAC | AAGAGACAGGAAACCCTCGG |
| *Ptgs2* | CGGTGAAACTCTGGCTAGACAG | GCAAACCGTAGATGCTCAGGGA |
| *Tf* | AGGCGCTTTCAGGTGTCT | CATCAAGGCACAGCAACTCA |
| *Ncoa4* | AGCTTTGCAGAGTGTGTGTG | ATGCTTCTCAGGCTCAGGTT |
| *Hmox1* | AAAGATTGCCCAGAAAGCCC | CTCCTCAAAGAGCTGGATGTTG |
| *Slc7a11* | TCCTGCTTTGGCTCCATGAACG | AGAGGAGTGTGCTTGCGGACAT |
| *Gpx4* | ACAAGAACGGCTGCGTGGTGAA | GCCACACACTTGTGGAGCTAGA |
| *Tp53* | GCGCACAGAGGAAGAGAATC | CCACGGATCTGAAGGGTGA |
| *Nrf2* | CACATCCAGTCAGAAACCAGTGG | GGAATGTCTGCGCCAAAAGCTG |
| *Vdac2* | AGGATTTGGTTTTGGGTTGGT | TGGTCTCCAAGGTCCCAGTA |
| *Nox1* | ATCCTTGGGTCAACATTGGC | CTCAGGAAGGACAGCAGATTG |
| *Gapdh* | AGCCACATCGCTCAGACAC | GCCCAATACGACCAAATCC |

**Supplement Figure S1.** The western blot image of Actin.

(a. Control of MDA-MB-231; b. ATR of BT-474; c. control of BT-474; d. ATR of MDA-MB-231)

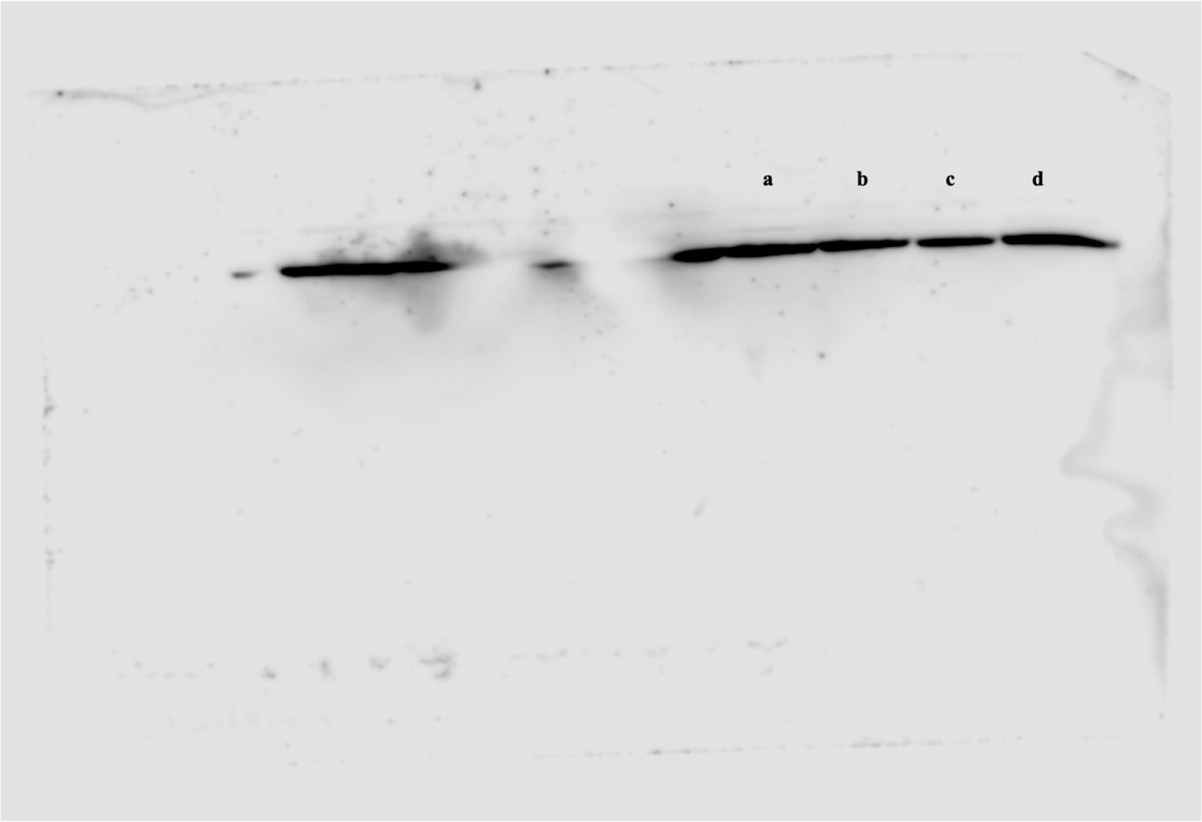

**Supplement Figure S2.** The western blot image of GPX4 in BT-474

(a. Control; b. ATR)

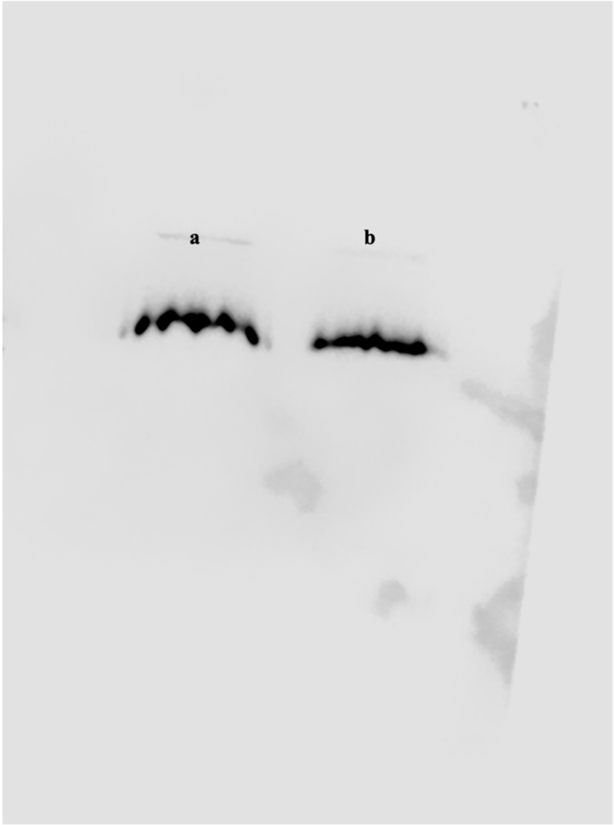

**Supplement Figure S3.** The western blot image of GPX4 in MDA-MB-231

(a. Control; b. ATR)

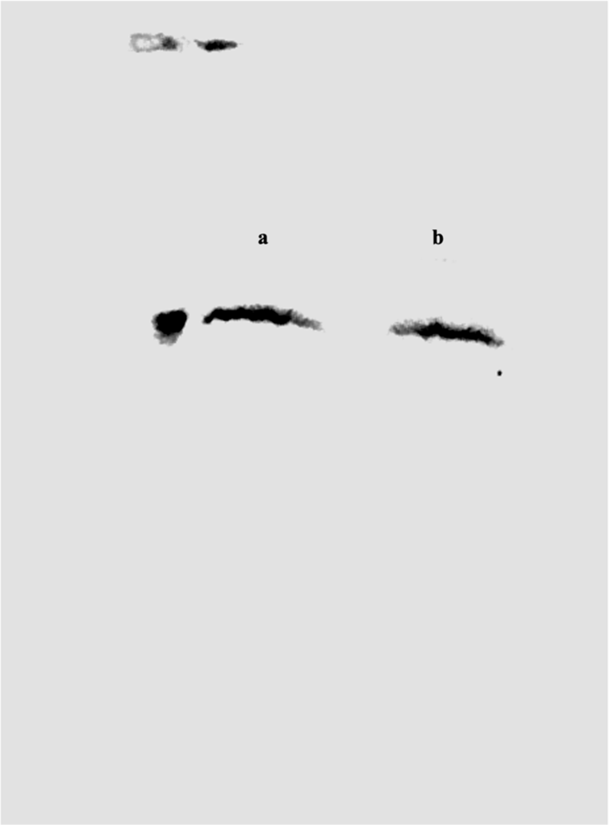

**Supplement Figure S4.** The western blot image of SLC7A11 in BT-474

(a. Control; b. ATR)

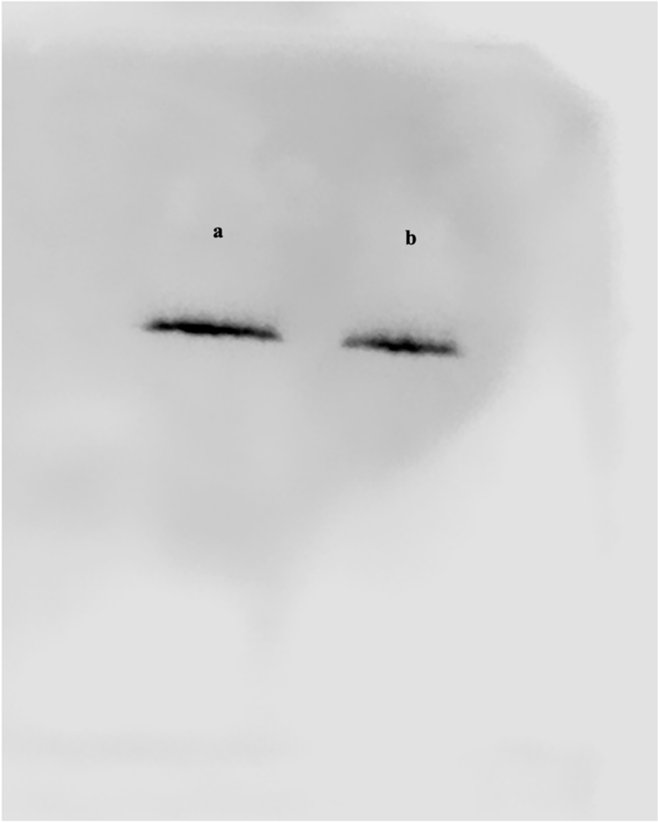

**Supplement Figure S5.** The western blot image of SLC7A11 in MDA-MB-231

(a. ATR; b. Control)

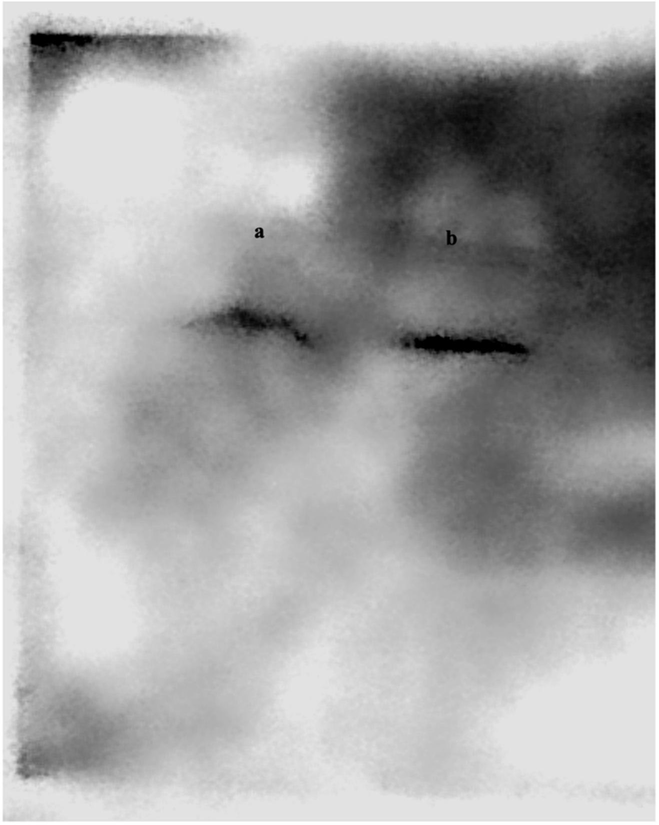

**Supplement Figure S6.** The western blot image of LPCAT3 in BT-474

(a. Control; b. ATR)

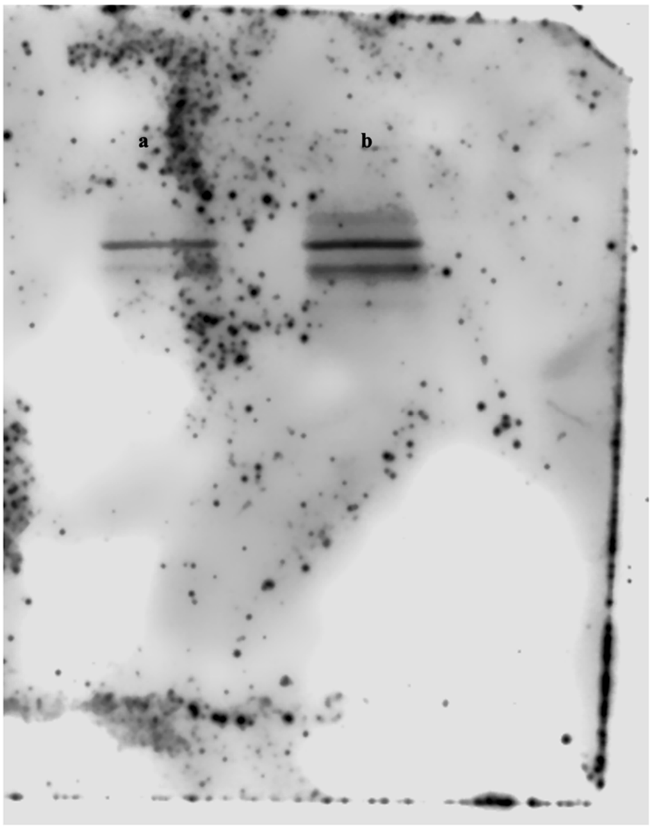

**Supplement Figure S7.** The western blot image of LPCAT3 in MDA-MB-231

(a. Control; b. ATR)

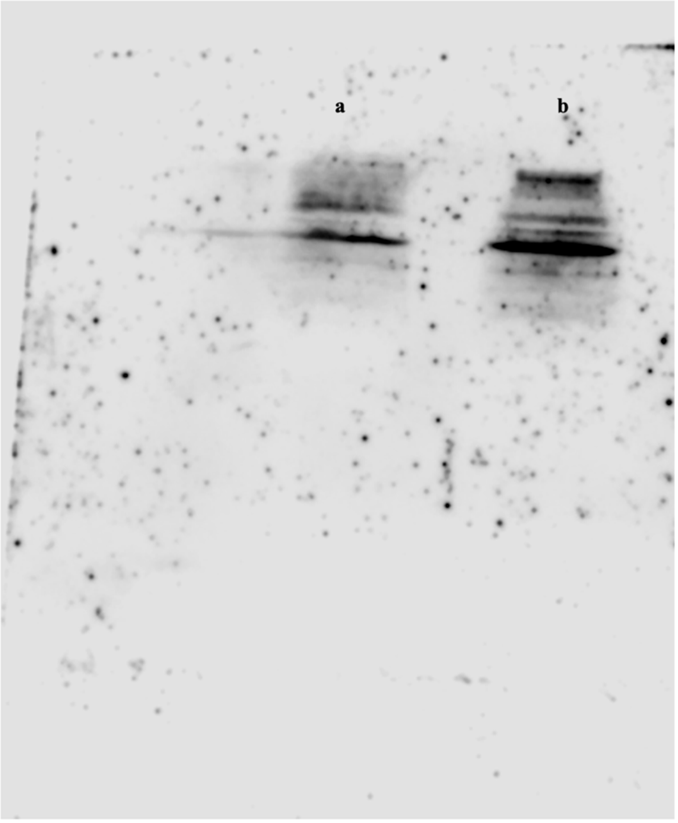

Supplement: Supplementary file 1 [file pharmaceuticals-17-01380-s001.zip › pharmaceuticals-3170133-supplementary.pdf]
